# Ten simple rules for effective use of generative AI for code development in environmental science

**DOI:** 10.1371/journal.pcbi.1014627

**Published:** 2026-08-17

**Authors:** Rachel A. King, Laurel Abowd, Carlo W. Broderick, LM Bradley, Max F. Czapanskiy, Mona M. Farnisa, Erica M. Ferrer, Carmen Galaz García, Darian Gill, Nicole M. Greco, Juliette Jacquemont, Justin A. Kadi, Li Kui, Gretchen LeBuhn, Liying Li, Abigail Meyer, Marisa Morse, Evan Patrick, Samantha Shanny-Csik, Nicholas A. C. Tucker, Zhe Wang, Caitlin R. Fong

**Affiliations:** 1 National Center for Ecological Analysis and Synthesis, University of California Santa Barbara, Santa Barbara, California, United States of America; 2 Bren School of Environmental Science and Management, University of California Santa Barbara, Santa Barbara, California, United States of America; 3 College of Creative Studies, University of California Santa Barbara, Santa Barbara, California, United States of America; 4 Marine Science Institute, University of California Santa Barbara, Santa Barbara, California, United States of America; 5 Department of Biology, San Francisco State University, San Francisco, California, United States of America; University of Colorado Boulder, UNITED STATES OF AMERICA

## Introduction

The explosion of generative artificial intelligence (GenAI, Table 1) into scientific disciplines is rapidly transforming how scientists conduct their research. The potential of GenAI to accelerate time consuming parts of the scientific process has led to its incorporation into nearly every aspect of research, from planning experiments to analyzing data and writing papers [1–4]. The benefits of using GenAI range from increasing productivity [5] to reducing language barriers [6] to scoping research directions [7]. However, emerging discrepancies in GenAI use across disciplines, age, career stage, demographics, and human development status [4,8] have led to concerns that instead of broadening participation, GenAI will exacerbate existing inequities in science [9]. Further concerns about the rigor and reproducibility of GenAI [10] have made some researchers skeptical about the effectiveness of using GenAI in scientific research. To avoid entrenching existing barriers and to ensure that researchers are using tools in ways that align with best research practices and ethical considerations, scientific communities must proactively develop shared norms and guidelines for the use of GenAI that are tailored to their disciplinary contexts.

**Table 1:** Definitions of key terms.

Term	Definition
Agentic AI	A class of AI system designed to perform complex, multi-step tasks by planning, delegating, and iteratively executing actions using tools and/or models to execute user goals. Agentic AI systems typically combine an LLM with external tools, memory, and control logic that enable goal-directed behavior and adaptive task decomposition, often with varying degrees of human oversight.
AI Agent	An AI agent is an instance of an agentic system that uses a large language model (or similar model) to interpret user instructions, select and use tools, and carry out sequences of actions within a defined environment. AI agents operate under explicit constraints and permissions, and their behavior is typically bounded by system rules, available tools, and human approval mechanisms rather than full autonomy or independent learning.
Context File	A plain text file (e.g., AGENTS.md) that provides persistent background information and instructions on how a GenAI tool or AI agent should respond to prompts and generate output.
Context Window	Similar to working memory, this determines how much text an LLM can remember at one time before needing to summarize it [16].
Generative Artificial Intelligence (GenAI)	A particular class of artificial intelligence algorithms trained to produce new content that can take the form of text, audio, video and imagery [17].
GenAI model	The underlying trained system (or checkpoint) used to generate outputs in a GenAI tool.
GenAI tool	The platform through which one interacts with a GenAI model, ranging from chatbots like ChatGPT [18] or Gemini [19] to interactive development environments (IDEs) like Cursor [20].
Large Language Model (LLM)	A type of deep learning model designed to respond to and generate natural language and other types of content. These models are trained on huge quantities of data to learn underlying patterns they then use to produce content based on what is statistically most likely to follow a prompt [16].

The field of environmental science could benefit from advances in GenAI tools to quickly develop insights and solutions to pressing environmental challenges like wildfire resilience, climate change, and biodiversity conservation [11,12]. However, as members of the environmental science community, we note that the adoption of GenAI in our field appears highly heterogeneous and unguided. Existing recommendations for GenAI use are typically geared toward science broadly [13] or coding for software engineers [14], which may not address the particular needs of environmental science researchers. Without sufficient resources to help guide researchers in the effective use of GenAI in their work, inequities and inefficient or questionable research practices could proliferate in the field.

As members of an environmental science research center that serves the broader scientific community, we have observed individuals who are hesitant to use GenAI, including some co-authors, because they doubt its effectiveness, feel unsure about how to maintain core principles of reproducibility and transparency, and have concerns about the environmental and societal impacts of GenAI. To address these concerns, we brought together our community, representing researchers, software developers, data analysts, and professors, to discuss and identify 10 simple rules for effective use of GenAI in coding for environmental science.

### Audience and process

We developed this guide for members of the environmental science community who use GenAI or are curious about using GenAI to write code and conduct data analyses while maintaining a high level of scientific rigor in their work. We all have backgrounds in various domains of environmental science and code on a regular basis, but we use GenAI to varying degrees (rarely to daily). Through a series of facilitated discussions, we identified important lessons learned through our own use of GenAI and subsequently streamlined these into 10 rules. We refined these rules through literature review and co-writing. Given the rapid development of research on GenAI, we frequently include references to pre-print articles and non-peer-reviewed material, such as reports or news articles, to provide timely perspectives on this topic. Our use of these sources should not unduly impact the key points of our narrative because rules were developed through direct user experience.

These rules focus on technical considerations and practices specific to GenAI use, especially where it creates novel challenges or requires new approaches, rather than broader best practices related to scientific rigor, open science, or coding. The rules further assume that an individual has determined that GenAI use is both appropriate and allowable for the identified task and do not describe the ethical considerations underlying that decision. Guidance on the ethical use of GenAI represents an active and complementary area for future research that requires interdisciplinary expertise beyond the scope of this paper [15]. However, we do discuss potential consequences of GenAI use that should be considered to avoid perpetuating social and environmental inequities.

## Rules

The rules emerging from our discussion generally fall into one of three categories, similar to those identified in Bridgeford et al. [14]: before, during, and after coding with GenAI. To best apply these rules, users should first understand that most GenAI systems operate through pattern recognition and not through true reasoning [21]. As a result, any code or statistical methods suggested by GenAI are the result of learned patterns in training data, not explicit testing of assumptions, verification of correctness, or awareness of context-specific constraints. This context helps users critically evaluate outputs; for example, recognizing that suggested models may violate statistical assumptions, or that code may appear plausible but incorrect. It also provides important context for ethical considerations. GenAI systems are trained on existing data and methods, therefore outputs reflect prior work without clear attribution, and propagate biases present in their training data [22]. Users should remain mindful of issues related to intellectual ownership, proper citation, and responsible use of generated content.

To ground each rule in practice, we draw on an existing data science project as a running example of each rule in Table 2: the Wildfire Resilience Index (WRI), an open-access tool designed to measure community and landscape resilience to wildfire. The WRI integrates heterogeneous data streams including satellite imagery, land-cover and vegetation traits, and socioeconomic variables, into a spatially explicit, multi-domain composite index intended to inform resource managers, decision makers, and communities. Developed by a six-person analytic team with subject matter expertise contributed by a broader working group spanning multiple institutions, the project required exactly the kind of complex, multi-contributor data synthesis GenAI tools are increasingly designed to support. We note that any tools referenced in the manuscript serve as examples and are not recommendations for any particular product.

**Table 2:** Examples of how the rules were applied to a real data science project, the Wildfire Resilience Index (WRI; https://www.wildfireindex.org/).

Rule	Application
Rule 1. Understand your domain (or take the time to learn it)	The project team was led by a PhD scientist with over 10 years of professional experience, with additional oversight of a senior scientist and a 10-member working group that provided the expertise required for such a complex project. The lead project scientist was responsible for every major decision and engaged in code review and output validation. No key decisions were made by AI.
Rule 2. Use a GenAI tool tailored to your task	Early in the project, team members mostly used general-purpose chatbots for broad exploration. As the project matured into a multi-contributor codebase spanning R and Python pipelines, the team shifted to an AI-native IDE (Cursor [20]) to maintain consistency across scripts, manage dependencies, and support automated refactoring.
Rule 3. Set up your GenAI platform for your task	The set-up of GenAI tools evolved as the WRI team gained experience in their use and project complexity increased. We moved from using reusable project descriptions and chatbot system prompts toward more structured context management systems that maintained project information across sessions. Specifically, the team created a shared context file describing the project’s spatial extent, the structure of the index, data source conventions, and preferred coding style in both R and Python. Providing this background at the start of each session meant that AI tools generated code consistent with existing pipeline conventions rather than producing generic solutions that required extensive reworking. As the project matured, we started using OpenClaw [59] to build a shared workspace that maintains project context across sessions. Rather than re-briefing the AI at the start of each coding session, the team co-developed a set of structured context files iteratively with AI: SOUL.md encodes the project’s scientific values and how the agent should reason about wildfire resilience; IDENTITY.md is a quick-reference card with project name, role, and metadata; AGENTS.md defines procedural rules for common workflows like data ingestion and domain score aggregation; USER.md captures individual researcher roles and preferences so outputs are tailored to each contributor; TOOLS.md documents the R and Python environments and key packages; HEARTBEAT.md schedules recurring checks such as verifying pipeline integrity or flagging when new climate or land cover data becomes available; and BOOTSTRAP.md runs once at setup to configure the agent’s identity for the project. These files enabled agents to start each session knowing the project’s spatial extent, domain structure, coding conventions, and team norms without any manual re-briefing.
Rule 4. Give GenAI tools only as much access as needed	The team worked on a shared high-performance computing system where multiple projects and users share compute resources. When using AI agents, the team scoped agent access to only the relevant project directories and excluded sensitive paths (e.g., home directories, credential stores, and shared system locations) using configuration-based controls such as ignore rules and restricted workspace settings. While fully isolated virtual machine environments could further reduce system-level risk, they are not used in this context due to the need for direct access to high-performance computing resources, shared file systems, and cluster tooling required for large-scale analysis. Instead, the team relied on least-privilege scoping within the live environment, ensuring the agent could only read and modify files necessary for the task while limiting broader system access. This reduced the risk of unintended changes to unrelated parts of the system while preserving access to essential compute infrastructure.
Rule 5. Adhere to principles of data privacy and security	The team works exclusively with publicly available data such as satellite imagery, census variables, climate model outputs, and species distribution data and therefore faces minimal data privacy risk when using GenAI tools. If extensions to this work require sensitive data, such as Indigenous community data, private community member data, unpublished agency datasets, or fine-grained socioeconomic variables or survey data, the team would adjust the workflow to process data locally or in a folder not visible to AI tools with code generated manually. If coding assistance were needed, the creation of dummy variables and simulated data would be used to generate code, followed by porting it to a location without AI access to run scripts to generate data layers.
Rule 6. Manage long GenAI conversations	Team members noticed that during long exploratory sessions in which the target goal shifted, the model began suggesting code and fixes that ignored earlier outputs and produced complex workarounds. When directly probed, the model identified context rot as the issue – it had forgotten previous attempted solutions. Starting a fresh session with a concise summary of prior decisions restored output quality.
Rule 7. Verify and validate GenAI output	Validation was a continuous, multi-layered process throughout project development. The team generated extensive visualizations at every stage, for example, distributions of domain scores, maps of intermediate outputs, and cross-variable comparisons to catch anomalies before they propagated downstream. Handling missing data was a recurring challenge: NA values in data layers required explicit decisions about imputation, masking, and edge cases, all of which were tested deliberately rather than assumed. The team also used multiple GenAI models for cross-validation, drafting code in one model and having another critique the logic and assumptions. Internal peer review among team members added a human layer of domain-grounded scrutiny. Throughout, GenAI accelerated the validation process itself by generating summary tables and interactive maps, flagging distributional outliers, and annotating functions, making rigorous checking faster and more tractable than it would have been otherwise.
Rule 8. Document your process	The WRI team documented their process at multiple levels. README files in the project repository recorded data source conventions, pipeline structure, and coding standards. GitHub issues served as a living log of analytical decisions and captured what was decided and why, including justifications for domain weighting choices and how edge cases were resolved. A project manager maintained notes on key decisions made during team meetings, creating an auditable record that did not rely on an individual’s memory or on GenAI chat histories. Finally, the team created an extensive supplementary document alongside the index describing all data choices and methodological rationale, a resource that serves both transparency and reproducibility for the broader research community.
Rule 9. Leverage GenAI to follow best practices	GenAI was transformative for code quality in the project in ways the team did not anticipate. Rather than simply generating new code, GenAI tools standardized syntax and style across a multi-contributor R and Python codebase, untangled spaghetti dependencies, and ensured that modular components worked together consistently. The team wrote down the best practices they wanted to follow, and AI implemented them uniformly across the entire pipeline quickly. The same process would have taken months to do manually across a medium-sized team. Annotation improved dramatically: functions that would previously have had minimal or no documentation were returned with clear, consistent comments. GenAI code review also caught insidious issues like hard-coded values and flagged them for correction before they could affect results. The team’s use of GenAI created a cleaner, more accessible, and more reproducible codebase than they would have produced without GenAI assistance.
Rule 10. Prepare for a minimal coding future	The WRI team invested significant time in figuring out which tools to use, how to configure them effectively, how to prompt well, and how to build and maintain workspace infrastructure. This investment allowed the team to glimpse what environmental science looks like when coding is no longer the bottleneck. The team recovered hours from debugging, dependency management, and code standardization they could redirect toward doing science: asking better questions, interrogating the data more deeply, and accelerating the path from analysis to real-world impact.

## Before

### Rule 1. Understand your domain (or take the time to learn it)

Effective use of GenAI tools requires understanding a task well enough to clearly articulate it, which draws on both subject matter expertise and knowledge of appropriate methods. When users lack the necessary expertise, they should make a deliberate effort to build it or collaborate with subject matter experts to ensure they can guide GenAI toward correct implementations. The risks of using GenAI without this foundation range from relatively innocuous time wasting seen in junior programmers [23] to substantial methodological errors that influence critical decisions [24]. For example, subtle analytical choices made in ecological modeling (e.g., species distribution modeling), data analysis and bioinformatics pipelines, and data cleaning can alter conclusions and lead to incorrect inferences [25–29]. Left undetected, those mistakes could carry serious consequences for end users. Notably, GenAI can help motivated users identify and fill in knowledge gaps to prevent such consequences (e.g., breaking down complex topics, creating study guides [30]).

### Rule 2. Use a GenAI tool tailored to your task

Selecting the right GenAI coding tool requires matching its capabilities to the security needs, scale, and complexity of your work. If tasks involve sensitive data, proprietary code, or other legal considerations, private or self-hosted tools that do not transmit inputs to external servers may be needed. Beyond that constraint, different GenAI tools have strengths and limitations that may suit different project stages. For instance, general-purpose chatbot interfaces, such as ChatGPT [18] and Google Gemini [19], may serve early ideation (e.g., brainstorming analysis strategies) and provide simple coding assistance (e.g., explaining unfamiliar error messages, drafting pseudocode). With more code-intensive tasks, using platforms with built-in GenAI agents (e.g., Posit AI within Positron [31]) provides a familiar coding interface while adding access to more advanced GenAI assistance. As project complexity grows, AI-native IDEs, such as Cursor [20], Devin Desktop (formerly Windsurf) [32], or GitHub Copilot [33], become increasingly valuable. Thanks to their ability to draw from an entire codebase, they provide context-aware code completion, multi-file edits, and inline chat grounded in your actual project files, which helps with tasks such as automated refactoring and bug fixing [34–36]. In all cases, the specific model used (e.g., Gemini 3.5, GPT-5) also influences performance, as models vary in the size of their context window, speed of analysis, and resource usage [37]. Because the GenAI landscape is evolving rapidly, the tools named here reflect the state of the field at the time of writing; the enduring principle is to match the tool to the task, not to adopt any particular product.

### Rule 3. Set up your GenAI platform for your task

Deliberate setup of GenAI tools for a specific task improves their performance and efficiency [38] and streamlines communication between you and the AI tool [39]. Describing the goals and providing background information (e.g., the problem setting, data, constraints, and desired output) improves the consistency and reliability of generated output (see Broderick [40] for an example setup). Depending on the complexity of the task and capabilities of the GenAI tool, the necessary setup can vary substantially. For beginner users, setup may be as simple as creating a reusable project description in a chatbot interface, while more advanced users of GenAI tools may benefit from hierarchical directories of context files for agentic workflows. Users should carefully consider what to include in context to ensure security of sensitive information (e.g., through .ignore files). Proper customization can also heighten reproducibility and transparency of analysis pipelines and complicated code bases when setup files encapsulate the appropriate background information.

## During

### Rule 4. Give GenAI tools only as much access as needed

The use of AI agents can introduce significant risks because these agents often inherit the same permissions and system access as the user and have been observed to enact adversarial and unexpected behaviors [41,42]. Some recommended practices to minimize risks include: running agents within isolated environments (e.g., virtual machines, containers, or dedicated systems), enforcing restricted permissions (such as through separate non-administrative user accounts), and ensuring secure, authenticated interactions with external tools and resources [43]. Importantly, these practices have restrictions enforced by the system surrounding the AI, rather than relying on the model itself, since language models are unreliable security gatekeepers [44]. Underlying these recommendations is the security principle of least privilege: an AI system should be granted the minimum access necessary. When in doubt, start with the most restrictive access that still allows the task to be completed, and expand only as needed.

### Rule 5. Adhere to principles of data privacy and security

GenAI usage complicates data confidentiality and carries leakage risk that users may underestimate [45]. Under standard consumer accounts, user prompts and outputs may be retained and incorporated into AI training pipelines, creating potential exposure [46,47]. Even where contractual agreements contain data in a controlled institutional ecosystem, prompts and outputs can be subject to legal discovery [48]. Several measures can reduce exposure, including the use of anonymized data, deployment of local GenAI models (e.g., LLaMA), and restricting access to sensitive data and folders (e.g., password protection). Users of GenAI should pay particular attention to these measures when working with sensitive or private data, such as those from Indigenous communities [49]. Ultimately, users bear the responsibility to uphold rigorous privacy and security standards.

### Rule 6. Manage long GenAI conversations

Long GenAI conversations that use more of a model’s available context window degrade model accuracy, a phenomenon known as “context rot” [50,51]. This happens because the self-attention mechanism that governs the model becomes less reliable as the number of tokens increases, raising the risk of “hallucinations”. Mitigation techniques to maintain accuracy include keeping chats short and focused and initiating new chats or sessions following changes in tasks and scope. For long-horizon tasks, compaction (i.e., condensing information size while maintaining key details from context), structured note-taking, and multi-agent architectures can help manage context effectively [52].

## After

### Rule 7. Verify and validate GenAI output

GenAI tools can both introduce and catch errors in code, which makes deliberate validation practices essential. Code produced by GenAI tools may execute cleanly but incorrectly [53], so users should verify outputs at multiple stages to catch issues early. Appropriate validation may include checking that data is read in accurately, testing edge cases informed by domain expertise, and confirming that intermediate results align with expectations. GenAI can accelerate this process: cross-model review (e.g., writing in one tool, reviewing in another) provides an independent check, while requesting on-demand visualizations or summary tables can surface problems that are hard to catch by reading code alone. GenAI also lowers the barrier to follow good coding practices that support validation [54,55], such as modular code with discrete, testable steps and well-annotated functions [56].

### Rule 8. Document your process

GenAI usage can obscure the decision-making process behind an analysis when key assumptions, decision pathways, and iterative refinements live only in chat histories that are difficult to trace and may be lost entirely. To counter this risk, users should document critical decisions directly in their code base through comments, README files, or version-controlled decision logs, akin to lab notebooks in experimental fields, ensuring that the reasoning behind analytical choices is preserved independently of any GenAI tool. In fact, GenAI can assist in this process by logging key decisions and justifications and saving these, such as in a markdown (.md) file. Configuration files that shape the behavior of GenAI, including system prompts, custom instructions, or project knowledge files, can also be version controlled and shared in repositories as part of the analytical record and to help others build on the user’s approach. When chat histories contain critical information not captured elsewhere, saving them to local, backed-up storage provides an additional safeguard.

### Rule 9. Leverage GenAI to follow best practices

GenAI tools can significantly improve adherence to best practices that people often struggle to maintain consistently, such as thorough documentation, style standardization across multi-contributor code bases, and well-organized data repositories. Tasks that are tedious or time-consuming to do manually, such as annotating functions, flagging hard-coded values, enforcing consistent coding standards, and writing comprehensive metadata, are well-suited to GenAI and can be applied uniformly across an entire project. Per rule 7, all AI-generated content should be reviewed and validated to ensure that it documents the project accurately. The result is cleaner, more auditable, and more reproducible work—not because the standards have changed, but because GenAI dramatically lowers the cost of meeting them.

### Rule 10. Prepare for a minimal coding future

Limited coding proficiency has long been a barrier to research in environmental science [57]. GenAI is shifting the bottleneck from programming toward scientific thinking, research design, and methodological rigor. Going forward, the ability to clearly communicate goals, frame problems, and effectively direct GenAI systems may well be more valuable than programming fluency for research productivity [58]. To prepare for this shift, users of GenAI will benefit from a continued focus on foundational skills, including a solid understanding of quantitative methods, data provenance, and research design, along with the ability to articulate scientific intent with precision. These skills are not new demands but fundamental needs to produce robust science efficiently with any tool, including GenAI.

## What could go wrong

In addition to discussing how individuals and communities can improve their GenAI coding practices, our workshop discussions surfaced a range of concerns stemming from the rapid adoption of GenAI in combination with a sparse regulatory environment. At their core, many of our concerns pointed to the same underlying issue: the risk that the transition to GenAI may narrow, rather than broaden, participation in environmental data science. Additional considerations related to the environmental impacts of expanding data centers to fuel the expansion of GenAI. We explore implications for both types of concerns below.

GenAI has been heralded as an equalizer by some, lowering barriers to entry and broadening participation. Yet, early research suggests that benefits accrue unevenly across experience levels, gender, and social context. One emerging pattern is that experience shapes how effectively users leverage GenAI. Within software development, entry-level developers used GenAI most frequently, but senior-level developers experienced greater benefits of AI adoption [60]. This pattern is paralleled in scientific computing, where students and less experienced programmers adopt GenAI at higher rates and simultaneously face disproportionately high consequences from over-reliance on and insufficient validation of GenAI [61]. This may result in a growing skills gap that accelerates broader changes across sectors that increasingly rely on GenAI.

Gender and wealth are two additional axes on which benefits of GenAI use appear to diverge. Male researchers tend to experience higher productivity gains and use GenAI more than their female counterparts, a pattern that has persisted across regions, sectors, and occupations [4,9]. Persistent underrepresentation of women using GenAI risks self-reinforcement, because systems trained on data that under-sample women may adapt more to the preferences, needs, and norms of men, potentially widening disparities in technology adoption and economic opportunity [9]. Beyond gender identity, patterns of wealth reveal additional barriers to GenAI adoption. A recent United Nations report found that while roughly two-thirds of people in some high-income countries use GenAI tools, usage in many low-income countries hovers near 5%—a staggering gap [8]. These patterns persist even within the wealthiest nation; in the United States, search interest in ChatGPT has been concentrated in coastal cities and depressed in rural regions including the Midwest, Appalachia, and the South, with county-level wealth emerging as a key predictor [62]. Taken together, these patterns suggest that the benefits of GenAI are not accruing equitably, but rather along familiar axes of social and economic inequality.

Not only are access disparities unlikely to resolve on their own, but the structural economics of the GenAI industry may actively deepen them as companies shift to a fee-based model [63]. Most leading GenAI companies (e.g., OpenAI, Anthropic) offer a tiered subscription model that locks the most powerful features behind paywalls. The shift to tiered pricing from free access will likely keep the most capable models out of reach of individuals at underfunded institutions, in low-income countries, or otherwise operating without institutional support. For scientists and coders in these circumstances, a pay-to-play model for GenAI does not democratize participation in science; rather, it perpetuates structural inequities. However, while the aforementioned trajectories of widening disparities are concerning, they are not predetermined outcomes. Realizing the potential of GenAI to help mitigate social inequalities will require both acknowledgement of existing inequities and deliberate policymaking to counteract the disparate harms GenAI could entrench [64].

A key issue among the policy challenges is the potential disruption GenAI could bring to the workforce, which raises deeper questions about institutional responsibility [65]. For decades, the technology industry has successfully advocated for large-scale public investment in computer science education, with the implicit promise that training more software developers would meet a robust and growing demand for their labor. The CHIPS and Science Act of 2022 alone authorized tens of billions for NSF workforce development in key technology areas, including AI and software, representing a substantial public commitment to building a pipeline of computing talent [66]. Yet, GenAI has disrupted that pipeline with striking speed. Unemployment among recent computer science and computer engineering graduates now exceeds 7.8% and 7%, respectively, higher than that of recent art history or English majors [67], while postings for software development roles fell by more than 70% between 2022 and 2025 [68]. As environmental science has become a more data-intensive field, often requiring advanced computational skills [69], the proliferation of GenAI could also alter the balance of jobs within this discipline. Although the data available to make predictions about the future is scarce, the current trend raises an uncomfortable question: if public funds built the pipeline, and private industry deployed the technology that undermined it, who bears responsibility for the workers left behind? Answering that question and designing policy responses equal to the disruption may be among the most consequential challenges GenAI poses at a systems level.

Finally, as environmental scientists, many discussions circled back to concerns about the potential environmental impacts resulting from the proliferation of GenAI. Many of these concerns are not unique to GenAI, but the newness of these tools makes comprehensive research quantifying their impacts sparse. There are, however, an array of social and environmental concerns across the life cycle of GenAI: from increased mining of rare earth metals for hardware components to noise pollution from cooling fans and the increased demand for electricity and water by data centers [70]. Energy use by data centers in the US is projected to represent 4%–12% of all electricity use by 2030 [71,72]. Cooling data centers requires large quantities of water, and by 2028 data centers are projected to use up to 32 billion gallons of water per year [73]. Limited federal regulations specific to data centers risk unsustainable development that could harm local communities and disproportionately impact marginalized communities if they follow trends similar to other pollutants [74,75]. There are opportunities to minimize the impacts, ranging from co-location of data centers with renewable energy sources to improving energy efficiency to leveraging reclaimed wastewater or other cooling methods to reduce water use [76]. Careful consideration of the environmental impacts of GenAI coupled with sound policies or regulations to encourage responsible development will be needed to avoid propagating harm on communities and ecosystems.

### Conclusions

GenAI is a powerful tool, but adopting it effectively for environmental science requires developing new skills and attending to considerations throughout the project that may be unfamiliar to many researchers. As a community, we are navigating a transition into this AI era together: learning, testing, adapting, and improving our skills with these new tools as we go. The 10 rules we present here offer a starting point for that process and complement existing standards that remain crucial to the transparency and rigorous practice of science [57]. When following the rules presented here, coding with GenAI can create rigorous and timely standalone products via transparent, reproducible, and accessible methods that adhere to our community principles. At the same time, organizations and institutions must recognize that without deliberate intervention, the benefits of GenAI may continue to accrue inequitably, maintaining or even widening existing disparities rather than lowering barriers to entry in the field. It is natural to feel skepticism or wariness toward any new tool, and we do not dismiss those concerns. Nevertheless, we have found it highly productive to engage openly with colleagues and explore what works, what does not, and what we do and do not want from these tools. The challenges GenAI poses for our field will not resolve themselves. Meeting them requires the kind of honest, sometimes difficult conversations that move a community forward. Ultimately, we have the power to shape how GenAI integrates into environmental data science, but only if we work proactively and collectively as a community towards that future.

### AI use statement

During the development of this manuscript, GenAI tools were used to facilitate writing and synthesis of ideas. Specifically, we used Elicit (https://elicit.com/) for portions of the literature review, Claude Opus 4.6 and OpenAI’s GPT-5.3 mini for editing and revising text. The authors identified the rules without input from GenAI.
